# Logopenic progressive aphasia with neologisms: a case report

**DOI:** 10.1186/s12883-019-1524-y

**Published:** 2019-11-25

**Authors:** Hiroyuki Watanabe, Manabu Ikeda, Etsuro Mori

**Affiliations:** 1 Department of Behavioral Neurology and Neuropsychiatry, Osaka University United Graduate School of Child Development, 2-2, Yamadaoka, Suita, Osaka, 565-0871 Japan; 2 Brain Function Center, Nippon Life Hospital, Osaka, Japan; 3 Department of Psychiatry, Osaka University Graduate School of Medicine, Suita, Japan

**Keywords:** Neurodegenerative disease, Primary progressive aphasia, Phonemic paraphasia, Verbal paraphasia, Neologism

## Abstract

**Background:**

Neologisms are commonly encountered in patients with acute cerebrovascular diseases, particularly in those with Wernicke’s aphasia. However, few studies have investigated primary progressive aphasia with neologisms in neurodegenerative disease.

**Case presentation:**

We describe the case of a 74-year-old, right-handed man who developed logopenic progressive aphasia (LPA) with neologisms. He was assessed with neuropsychological tests, magnetic resonance imaging, and single-photon emission computed tomography. Neologisms accounted for a relatively large portion of the paraphasic errors in the naming tests performed during the neuropsychological assessment. He had all the diagnostic features of LPA. Notably, the unique feature of this patient was the presentation of neologisms, which are seldom observed in typical LPA.

**Conclusions:**

Neologisms are considered rare symptoms in patients with early-stage LPA. Our findings in this case report provide new insights into the spectrum of clinical features in LPA.

## Background

Neologisms are non-words that are considered phonological errors when they share less than 50% of the phonemes with the target words [1, 2]. Phonological production deficit (conduction theory) [3], anomic error (anomia theory) [4], and their combination (two-stage error theory) [5] were posited as mechanisms for the development of neologisms. According to the conduction theory, neologisms, which are phonemic substitutions, are thought to be severe phonological transformations that distort the target words [3]. According to the anomia theory, neologisms result from the anomic condition and are substitutes for the root forms of lexical items when these root forms cannot be retrieved from the lexical system [4]. In addition, a “random syllable generator” has been proposed to characterize a mechanism within the anomia theory, in which novel words are created to fill the anomic gaps [6]. According to the two-stage error theory, neologisms result from a combination of phonemic and anomic errors [5]. However, it is difficult to examine the mechanism underlying neologism formation in a large cohort of patients with severe aphasia. Consequently, the underlying mechanisms of neologisms remain to be investigated [7].

Neologisms most commonly occur in patients with acute cerebrovascular disease, particularly in those with Wernicke’s aphasia. However, neologisms are rarely reported in progressive neurologic disorders, with fewer than five reported cases in the literature [8–11]. Primary progressive aphasia (PPA) is a collective term for neurodegenerative diseases with language impairment as the most salient feature. Logopenic progressive aphasia (LPA), a type of PPA, is a neurodegenerative syndrome characterized by word-finding difficulty, sentence-repetition deficits, phonological errors, and difficulty in comprehension associated with verbal short-term memory impairment [12]. Recent evidence indicates that neologisms occur in patients with advanced LPA [10, 11]. However, the theories accounting for neologisms in cerebrovascular disease have not been used to explain neologisms in neurodegenerative diseases. In this study, we investigated the potential cognitive mechanisms underlying neologisms in a patient with LPA.

## Case presentation

**Fig. 1:**
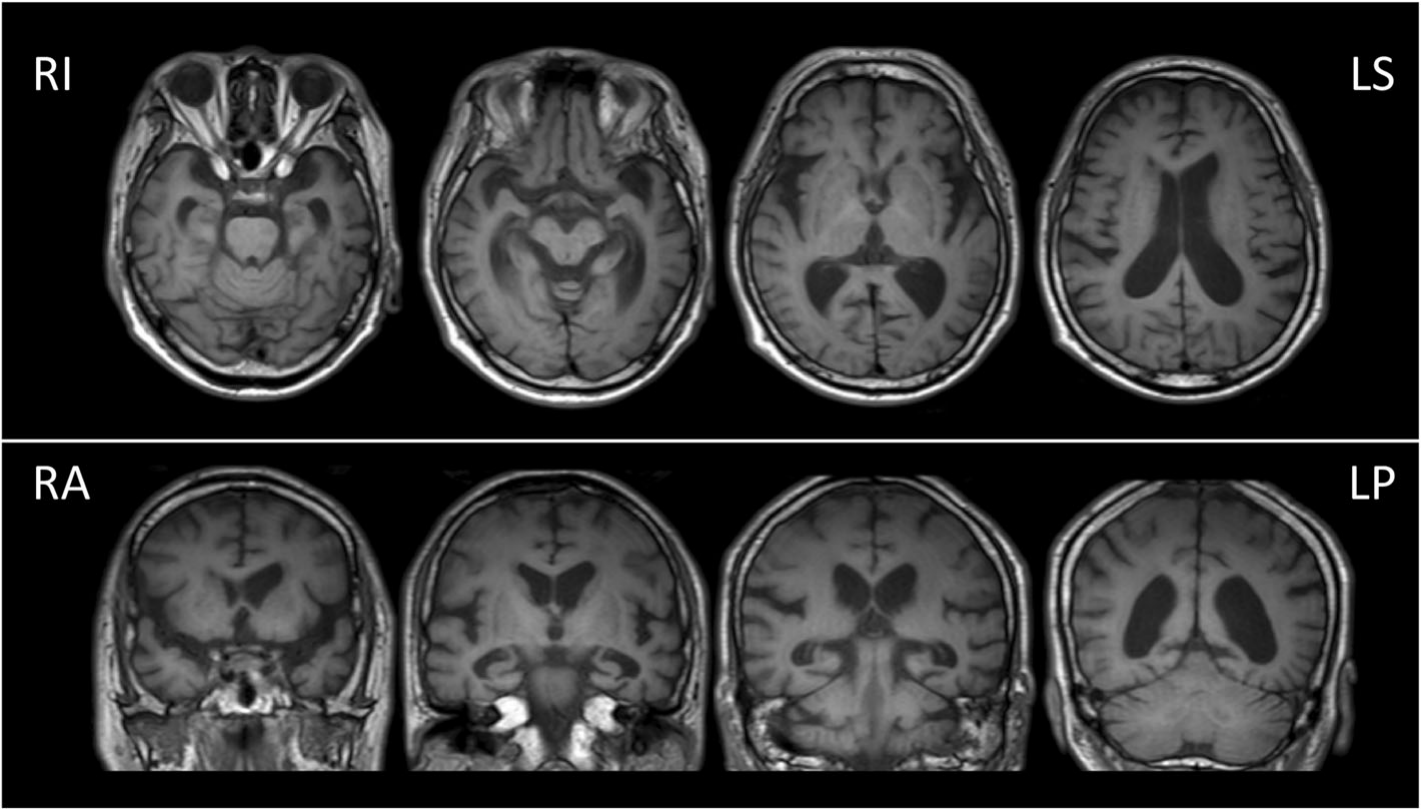
Brain magnetic resonance imaging. Brain magnetic resonance imaging (MRI) showing predominantly left-sided atrophy of the anterior and medial temporal lobe and enlargement of the left lateral ventricle. LP, left posterior; LS, left superior; RA, right anterior; RI, right inferior

**Fig. 2:**
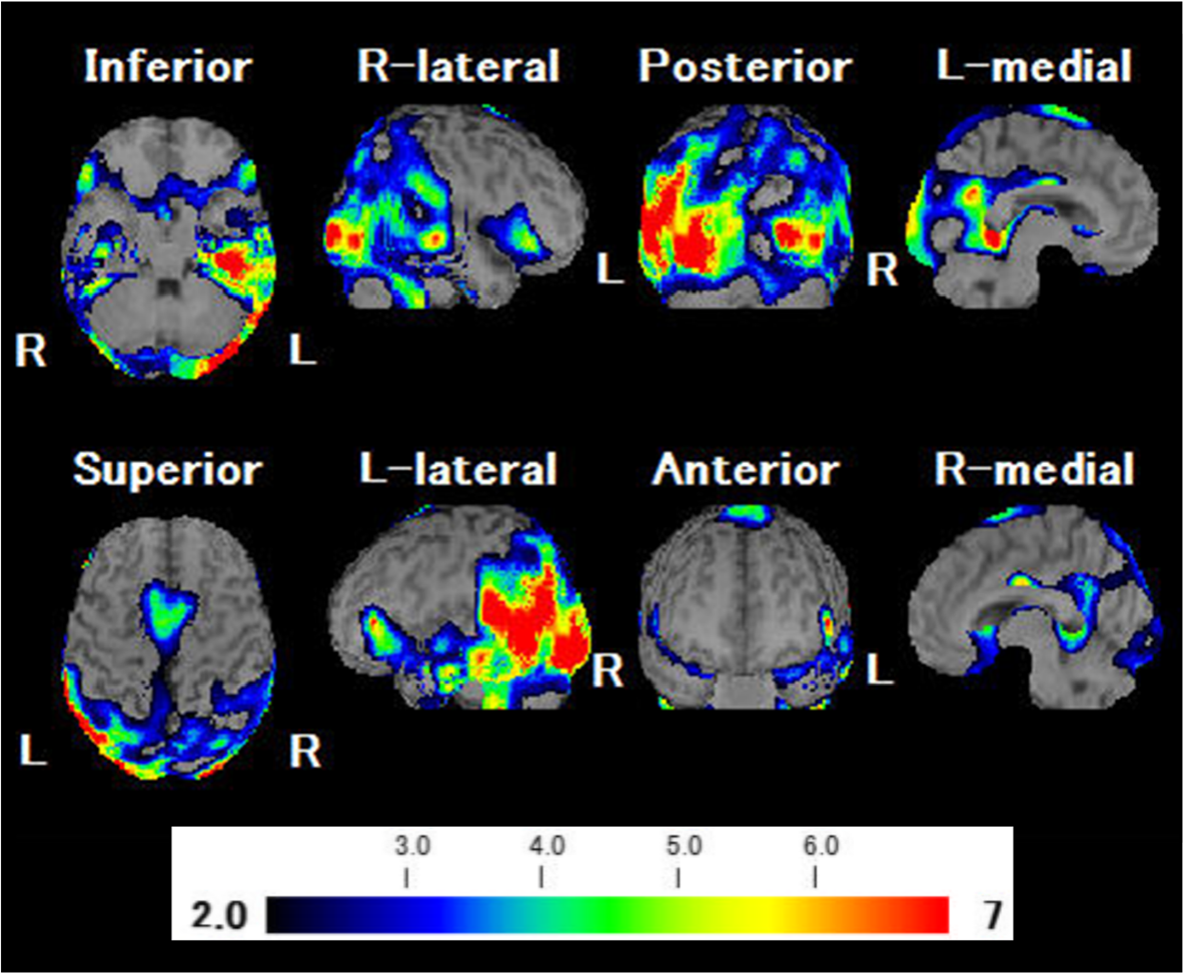
Brain single-photon emission computed tomography analysis with an easy Z score imaging system. *N*-iso-propyl-*p*-[^123^I] iodoamphetamine (IMP) single-photon emission computed tomography (SPECT) analysed with an easy Z score imaging system (eZIS) showing predominantly left-sided bilateral hypoperfusion of both the temporoparietal lobe and the posterior cingulate gyrus. L, left; R, right

A 74-year-old right-handed man visited Nippon Life Hospital with a complaint of slowly progressing difficulty in speaking and recognizing spoken words. He first experienced difficulty in speaking at the age of 71 years. Three years later, he started experiencing difficulty in recognizing spoken words. His medical history was unremarkable, and he was not on any medication. He had 12 years of education. He was fully conscious and oriented at the initial visit. No abnormalities were detected on physical examination, neurological examination, or routine laboratory tests. Brain magnetic resonance imaging (MRI) revealed predominantly left-sided atrophy of the anterior and medial temporal lobe and the parietal lobe and enlargement of the left lateral ventricle (Fig. 1). There was no evidence of hemorrhage or an ischemic lesion. *N*-iso-propyl-*p*-[^123^I] iodoamphetamine (IMP) single-photon emission computed tomography (SPECT) revealed predominantly left-sided bilateral hypoperfusion of the temporo-parietal lobes and the posterior cingulate gyrus (Fig. 2).

Neuropsychological evaluations were performed within 2 months after the initial visit. He retained awareness of his language impairments. Moreover, his motivation to communicate was well-preserved. On evaluation, his spontaneous speech was characterized by a slow rate and was limited to short utterances with frequent pauses due to significant word-finding difficulty. However, he had no apraxia of speech or agrammatism, and he was able to produce grammatically correct sentences without omission and/or misuse of grammatical morphemes. He exhibited a few phonemic paraphasias and a relatively large number of verbal paraphasias and neologisms. Neologisms in this study were classified as words sharing less than 50% of phonemes with the target words [1, 2]. Auditory comprehension in the spontaneous speech was mostly preserved, although he showed an inability to comprehend long and complex sentences.

**Fig. 3:**
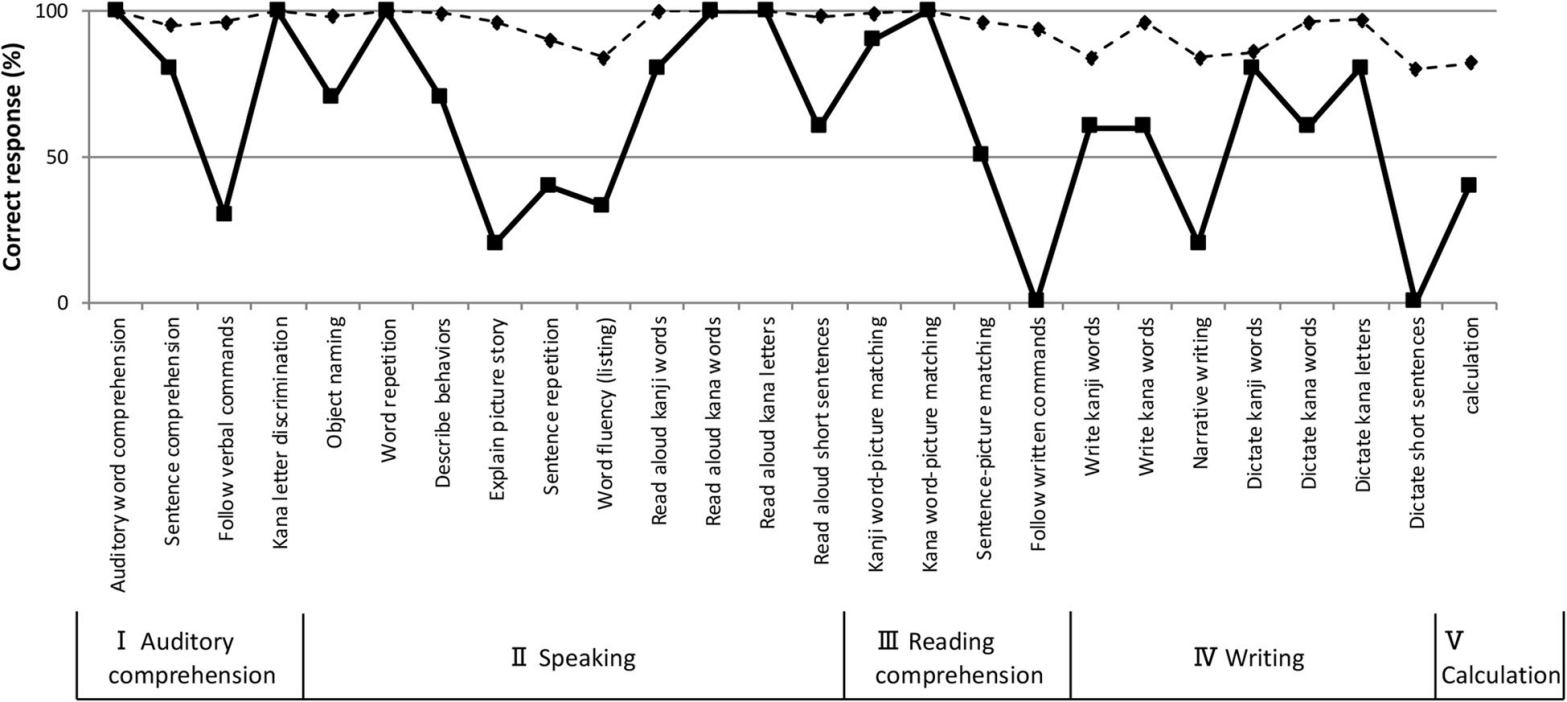
Profile of the Standard Language Test of Aphasia (SLTA). Solid line: the patient’s score; Broken line: mean score of 150 non-aphasic patients

A standard language test for aphasia (SLTA) [13] was performed within 2 months after the initial visit (Fig. 3). The SLTA, a comprehensive standardized battery test of language functions for adult Japanese speakers, is most commonly used in Japan. He provided correct responses for 8 out of 10 items in the *SLTA sentence comprehension tasks* [8/10 (80%) in our patient vs. a mean score of 9.5/10 (95%) in 150 non-aphasic individuals; Fisher’s exact test *p*-value = 0.5], indicating that his comprehension of short sentences was mostly preserved. In contrast, he provided correct responses for only 3 out of 10 items in the *SLTA following-verbal-commands tasks* [3/10 (30%) in our patient vs. a mean score of 9.6/10 (96%) in 150 non-aphasic individuals; Fisher’s exact test *p*-value = 0.003], indicating that his comprehension of long and complex sentences was impaired. He provided correct responses for 14 out of 20 items in the *SLTA object naming tasks* [14/20 (70%) in our patient vs. a mean score of 19.6/20 (98%) in 150 non-aphasic individuals; Fisher’s exact test *p*-value = 0.02], indicating that he had anomia. Few errors occurring during the *object naming tasks* were due to no responses (2/20, 10%) because of word-finding difficulty. During the evaluation, some paraphasias (4/20, 20%) occurred. Paraphasic errors during the *SLTA object naming tasks* were classified as verbal paraphasias (2/4, 50%), (e.g., “*isu*” ‘chair’ for “*tukue*” ‘desk’ and “*nemoto*” ‘root’ for “*torii*” ‘Shinto gateway’), and neologisms (2/4, 50%), (e.g., “*etoshima*” for “*enpitsu*” ‘pencil’ and “*hikoshiki*” for “*chochin*” ‘Japanese lantern’). With respect to neologisms, no responses resulted from the production of a phonemic error in a verbal paraphasia, [e.g., target = tomato and response = banona (phonemic error on banana)]. When he could not retrieve the target words, initial letter cues provided were ineffective.

To assess speech production ability, he was asked to tell a story about a four-frame cartoon in the *SLTA explanation-of-picture-story task*. A general score (range, 1–6) was generated based on the number of the included key words (“*aruku*” ‘walk’, “*boshi*” ‘hat’, “*tobu*” ‘fly’, and “*hirou*” ‘pick up’), grammatical errors, and character errors in this story. The obtained scores were defined as follows: 6, perfect sentences including four key words; 5, incomplete or paraphasic sentences including four key words; 4, sentences including three key words; 3, sentences including two key words; 2, sentences including only one key word; and 1, no key word. He scored a 2 in the *SLTA explanation-of-picture-story task*. Although the score of our patient was numerically considerably lower than the mean score of 150 non-aphasic individuals, the difference was not statistically significant [2/6 in our patient vs. a mean score of 5.8/6 in 150 non-aphasic individuals; Fisher’s exact test *p*-value = 0.061]. His speech was characterized by a slow rate and was limited to short utterances with frequent pauses due to significant word-finding difficulty. For example, when he was asked to tell a story, he only presented the following sentences: “… *kaze ga fuite boshi ga* … *michi de boshi wo* … *otoshite* ….” ‘… wind (particle) blow hat (particle) … street (particle) hat (particle) … drop ….’ ‘The wind blows the hat in the street. The hat falls.’ He had no apraxia of speech, paraphasia, omission, and/or misuse of grammatical morphemes.

He was only able to repeat two out of five items in the *SLTA sentence repetition task*. Although the score of our patient was considerably lower than the mean score of 150 non-aphasic individuals, the statistical difference was not significant [2/5 (40%) in our patient vs. a mean score of 4.5/5 (90%) in 150 non-aphasic individuals; Fisher’s exact test *p*-value = 0.167]. His difficulty in sentence repetition occurred in the latter part of the sentences. For example, when he was asked to repeat “*tonari no machi de kaji ga atta*” ‘a fire occurred in surrounding town’, he repeated only “*tonari no machi de* ….” Moreover, when he was asked to repeat “*watashi no ie ni inaka kara okina kozutsumi ga todoita*” ‘my parents sent a big package to my house’, he repeated only “*watashi no* … *inaka kara* ….” In addition, his digit-span performance was reduced (forward 3, backward 2). In contrast, his spatial span performance was normal (forward 6, backward 6). Therefore, these results revealed that our patient had verbal short-term memory impairment and repetition deficit. The scores of the *III Reading comprehension*, *IV Writing*, and *V Calculation tasks* were generally below the values of non-aphasic individuals, revealing deficits in each of these functions.

The Test of Lexical Processing in Aphasia (TLPA) [14], which is used widely in clinical settings in Japan, was performed within 2 months after the initial visit. The TLPA is the standard language test for individuals who speak Japanese, and a total of 200 items in line-drawing cards are included in the picture-naming task or the auditory word comprehension task. The number of correct responses during the TLPA picture-naming task was significantly low [79/200 (39.5%) in our patient vs. a mean score of 193.4/200 (96.7%) in 54 healthy individuals; chi-square value = 148.0; *p* < 0.0001]. Many errors occurring during the task were due to no responses [91/200 (45.5%)] because of word-finding difficulty. During the evaluation, various paraphasias [30/200 (15%)] occurred. The paraphasic errors were classified as phonemic paraphasia [1/30 (3%); e.g., “*nodabotoke*” for “*nodobotoke*” ‘Adam’s apple’], verbal paraphasias [23/30 (77%); e.g., “*daidokoro*” ‘kitchen’ for “*genkan*” ‘entrance’], and neologisms [6/30 (20%); e.g., “*gagato*” for “*koppu*” ‘glass’ and “*gochihara*” for “*chiritori*” ‘dustpan’]. Regarding neologisms, no response resulted from the production of a phonemic error in a verbal paraphasia. When initial letter cues were provided, only 13 out of 121 (10.7%) error words in the TLPA picture-naming task were converted to the correct words. Therefore, initial letter cues were mostly ineffective.

The auditory word comprehension task was performed using the same line-drawing cards as in the TLPA picture-naming task. In the TLPA auditory comprehension task, after listening to a spoken word, our patient was asked to match 1 of the 10 line-drawing cards. The number of correct responses in the TLPA auditory word comprehension task was slightly decreased [166/200 (83%) in our patient vs. a mean score of 199.4/200 (99.7%) in 53 healthy individuals; chi-square value = 33.2; *p* < 0.0001], indicating that his single-word comprehension was impaired, although it was mostly preserved in the context of conversational language use. In total, 26 out of 34 (76.5%) wrong words in the auditory word comprehension task could not be properly retrieved in the picture-naming task, indicating two-way anomia, i.e., difficulties in both word finding and word recognition for the same target word, e.g., target = tomato, response = difficulties in naming a “tomato” and auditory comprehension of “tomato” [15]. These findings suggested that the patient had semantic memory impairment.

Moreover, the word-repetition task was performed using the same words as those in the picture-naming and auditory word comprehension tasks in the TLPA. The number of correct responses for the word repetition task was nearly perfect [196/200 (98%); no data in healthy individuals], and there were only four phonemic paraphasias during the repetition task. Therefore, phonemic paraphasia seldom occurred during repetition, and the finding was in accordance with his spontaneous speech and performance in the naming and explanation-of- picture-story tasks.

Although the patient could not be directly evaluated due to aphasia, no prominent amnesia or attentional/executive dysfunctions were observed in his daily life. Because he was able to copy a Necker cube drawing, he had no prominent visuospatial dysfunction. He had no abnormality in praxis and showed excellent capability in imitation, pantomiming, and using tools with either hand. Thus, he exhibited no problems (except for language impairments) when performing activities of daily living.

## Discussion and conclusions

Based on the results of the assessment, this patient appears to meet the criteria for LPA, in which repetition deficits and word-finding difficulties in spontaneous speech and naming tasks are the core features [12]. His spontaneous speech was characterized by a slow rate with frequent pauses due to word-finding difficulty, but he had no apraxia of speech or agrammatism. In addition, he exhibited only a few phonemic paraphasias and relative sparing of single-word comprehension. He showed no problems in everyday life, except for aphasia and acalculia. Regarding the image findings, MRI revealed predominantly left-sided atrophy of the anterior and medial temporal lobe and the parietal lobe. In addition, SPECT imaging found predominantly left-sided, bilateral hypoperfusion of the temporoparietal junction area, which is mostly consistent with the patterns of LPA. Thus, he met all the diagnostic criteria for LPA. Notably, the unique feature of this patient was the presentation of neologisms, which is seldom observed in typical LPA.

Neologisms are likely to occur in LPA based on the conduction theory or two-stage error theory because phonemic paraphasia is one of the distinguishing characteristics of LPA [12]. However, to our knowledge, few studies have investigated LPA with neologisms. Only two cases [10, 11] of LPA have been reported in the literature. One patient in Caffarra’s report [10] and another patient in Funayama’s report [11] exhibited worsening aphasia and neologisms after LPA onset. Rohrer et al. [8] described a patient with PPA who exhibited impaired sentence repetition and severe auditory word comprehension at the first assessment, although this patient was not diagnosed with LPA, and in the following year, this patient developed a spoken jargon with neologisms. Phonological production deficits may be less severe in the early stages in neurodegenerative diseases than in cerebrovascular diseases involving critical language networks, particularly Wernicke’s aphasia. Therefore, neologisms that occur in patients with LPA in the early stage may be considered a relatively rare symptom.

The conduction, anomia, and two-stage error theories could be used to account for the occurrence of neologisms. According to the conduction and two-stage error theories, a phonological production deficit is considered to be involved in the occurrence of neologisms. According to the conduction theory, neologisms are considered severe phonological transformations [3]. However, phonemic paraphasia seldom occurred in this patient. Therefore, the severity of his phonological production deficit was mild. In addition, according to the two-stage error theory, neologisms are thought to result from a combination of phonemic and anomic errors [5]. However, no neologisms resulted from the production of a phonemic error in his verbal paraphasia. Based on these results, the origin of his neologisms could not be fully explained by the conduction theory or two-stage error theory. With regards to anomic condition, verbal paraphasias accounted for > 70% of his paraphasic responses during the TLPA. Moreover, initial letter cues were generally ineffective for wrong words retrieved in the TLPA picture-naming task, and two-way anomia accounted for > 70% of the wrong words in the TLPA auditory word comprehension task. These TLPA results indicated that our patient had a semantic memory impairment, which has been observed in patients with semantic dementia [15]. Therefore, his neologisms may be explained by the anomia theory. However, neologisms are rarely reported in semantic dementia. Thus, the anomia theory alone may not be enough to explain the etiology of language deficits leading to the production of neologisms in patients with neurodegenerative disease. Accordingly, further investigations are needed to discern this issue.

The present study has several limitations. First, although neologisms accounted for a relatively large portion of the paraphasic errors in the SLTA and the TLPA naming tasks, only a few neologisms were observed in our patient. Potential cognitive mechanisms might underlie neologisms in the patient with LPA in this study. Further studies with a large sample size are needed to determine the valid theories accounting for neologisms in neurodegenerative diseases. Second, we observed no statistically significant difference in the patient’s speech production ability (as assessed using the *SLTA explanation-of-picture-story task*) or sentence repetition ability (as assessed using the *SLTA sentence repetition task*), although the score of our patient was numerically considerably lower than the mean score of non-aphasic individuals. We speculate that the nonsignificant differences resulted from the extremely limited potential ranges for scores on these tests (i.e., ranges of 1–6 and 0–5 for the *SLTA explanation-of-picture-story* and *sentence repetition tasks*, respectively), and we therefore believe that our patient’s speech fluency levels and sentence repetition abilities were not equal to those of non-aphasic individuals. Third, pathological examinations were not conducted in the present study. Our patient had semantic memory impairment and anterior temporal lobe atrophy based on MRI findings. The findings indicate that the patient might have semantic dementia, although neologisms rarely occur in semantic dementia.

In this case report, we describe a patient with LPA who presented with neologisms, which are seldom observed in typical LPA. Neologisms may be considered a relatively rare symptom in patients with LPA in the early stage. Our findings in this case report provide new insights into the spectrum of clinical features of LPA.

## Data Availability

All data generated or analyzed during this study are included in this published article.

## Abbreviations

IMP: *N*-iso-propyl-*p*-[^123^I] iodoamphetamine
LPA: Logopenic progressive aphasia
MRI: Magnetic resonance imaging
PPA: Primary progressive aphasia
SLTA: Standard language test for aphasia
SPECT: Single-photon emission computed tomography
TLPA: Test of Lexical Processing in Aphasia
